# The conserved mosaic prophage protein paratox inhibits the natural competence regulator ComR in *Streptococcus*

**DOI:** 10.1038/s41598-018-34816-7

**Published:** 2018-11-08

**Authors:** Lauren Mashburn-Warren, Steven D. Goodman, Michael J. Federle, Gerd Prehna

**Affiliations:** 1 Center for Microbial Pathogenesis, The Research Institute at Nationwide Children’s Hospital, Columbus, Ohio USA; 2 Department of Medicinal Chemistry and Pharmacognosy, Center for Biomolecular Sciences, University of Illinois at Chicago, Chicago, Illinois USA; 3 Department of Microbiology, University of Manitoba, Winnipeg, Manitoba Canada

## Abstract

Horizontal gene transfer is an important means of bacterial evolution. This includes natural genetic transformation, where bacterial cells become “competent” and DNA is acquired from the extracellular environment. Natural competence in many species of *Streptococcus*, is regulated by quorum sensing via the ComRS receptor-signal pair. The ComR-XIP (mature ComS peptide) complex induces expression of the alternative sigma factor SigX, which targets RNA polymerase to CIN-box promoters to activate genes involved in DNA uptake and recombination. In addition, the widely distributed *Streptococcus* prophage gene paratox (*prx*) also contains a CIN-box, and here we demonstrate it to be transcriptionally activated by XIP. *In vitro* experiments demonstrate that Prx binds ComR directly and prevents the ComR-XIP complex from interacting with DNA. Mutations of *prx in vivo* caused increased expression of the late competence gene *ssb* when induced with XIP as compared to wild-type, and Prx orthologues are able to inhibit ComR activation by XIP in a reporter strain which lacks an endogenous *prx*. Additionally, an X-ray crystal structure of Prx reveals a unique fold that implies a novel molecular mechanism to inhibit ComR. Overall, our results suggest Prx functions to inhibit the acquisition of new DNA by *Streptococcus*.

## Introduction

Bacterial evolution is driven by the acquisition of new genetic material through the processes of conjugation, transduction, and natural competence. Conjugation is a direct transfer of DNA between bacteria^1^, transduction is the acquisition of new genetic material through infection by bacteriophage^2^, and natural competence is the ability of bacteria to acquire DNA directly from the extracellular environment^3^. In Gram-positive bacteria, natural competence is regulated by peptide pheromone dependent quorum sensing (QS) systems^4,5^. In particular, *Streptococcus species* use either the ComCDE or ComRS QS system to regulate the expression of SigX, an alternative sigma factor. SigX recognizes a specific DNA sequence (TACGAATA) known as a CIN-box, which directs the core-RNA polymerase to initiate transcription of several genes having a CIN-box within their promoter region. These genes are known as “late” competence genes and include those that encode the DNA uptake machinery and enzymes used in recombination of acquired DNA^6–8^.

*Streptococcus pyogenes* (Group A Streptococcus or GAS) utilizes an Rgg-like transcriptional regulator known as ComR, and a small peptide encoded immediately downstream of *comR* known as XIP (*sigX*-inducing peptide), to regulate genes associated with natural competence^9,10^. XIP is derived from ComS, a small 17aa peptide that is secreted and proteolytically processed to become the mature 7-8aa active pheromone^10,11^. Upon uptake into the cell, XIP binds and activates ComR^10,12^, which induces a significant conformational change and dimerization that allows for the recognition of the *comS* (XIP) and *sigX* promoter regions^13,14^. This creates a positive feedback loop for the activation of the SigX regulon. Interestingly, *comRS* and all of the genes required for natural transformation are present in all pyogenic, mutans, bovis, and suis streptococci, although not all of these groups have been shown to be naturally competent. While a mechanism of silencing is currently unknown for ComRS in pyogenic streptococci, the closely related Rgg2/3 quorum sensing system of *Streptococcus pyogenes* is silenced by the secreted protease PepO that degrades the peptide pheromones^15^. In addition, competence in *Streptococcus pneumoniae* is rapidly turned off after induction by DprA by the disruption of CSP-dependent quorum sensing signal transduction^16^. Similarly, unique to *Streptococcus mutans*, a nested open reading frame within the *sigX* coding sequence encodes a 69aa protein called XrpA, which has a negative regulatory effect on competence induction^17,18^. Thus, it is likely that mechanisms to turn off ComRS-dependent competence development exist in other streptococci.

Although natural competence is an important aspect for the evolution of *Streptococcus*^3^, transduction by bacteriophages appears to be a driving force for streptococcal clonal diversification^19,20^. It is thought that horizontal gene transfer via transduction between different bacterial populations can be the most rapid mechanism for the evolution of a prokaryotic genome^2,21^. Additionally, bacteriophages are responsible for the dissemination of many virulence factors that are central to host pathogenesis^2,22^. For example, in the human pathogen *S*. *pyogenes*, toxins are encoded by genes located within the integrated prophage genomes. These phage-encoded toxins include superantigens (eg. SpeA)^23^, which are involved in GAS invasive diseases such as necrotizing fasciitis and streptococcal toxic shock syndrome, but their beneficial roles to *S*. *pyogenes* may extend to non-invasive interactions with the host^24,25^. GAS prophage genomes also encode DNases (e.g. Sda1)^26^, which are critical for the degradation of DNA released as neutrophil extracellular traps^27^.

**Figure 1:**
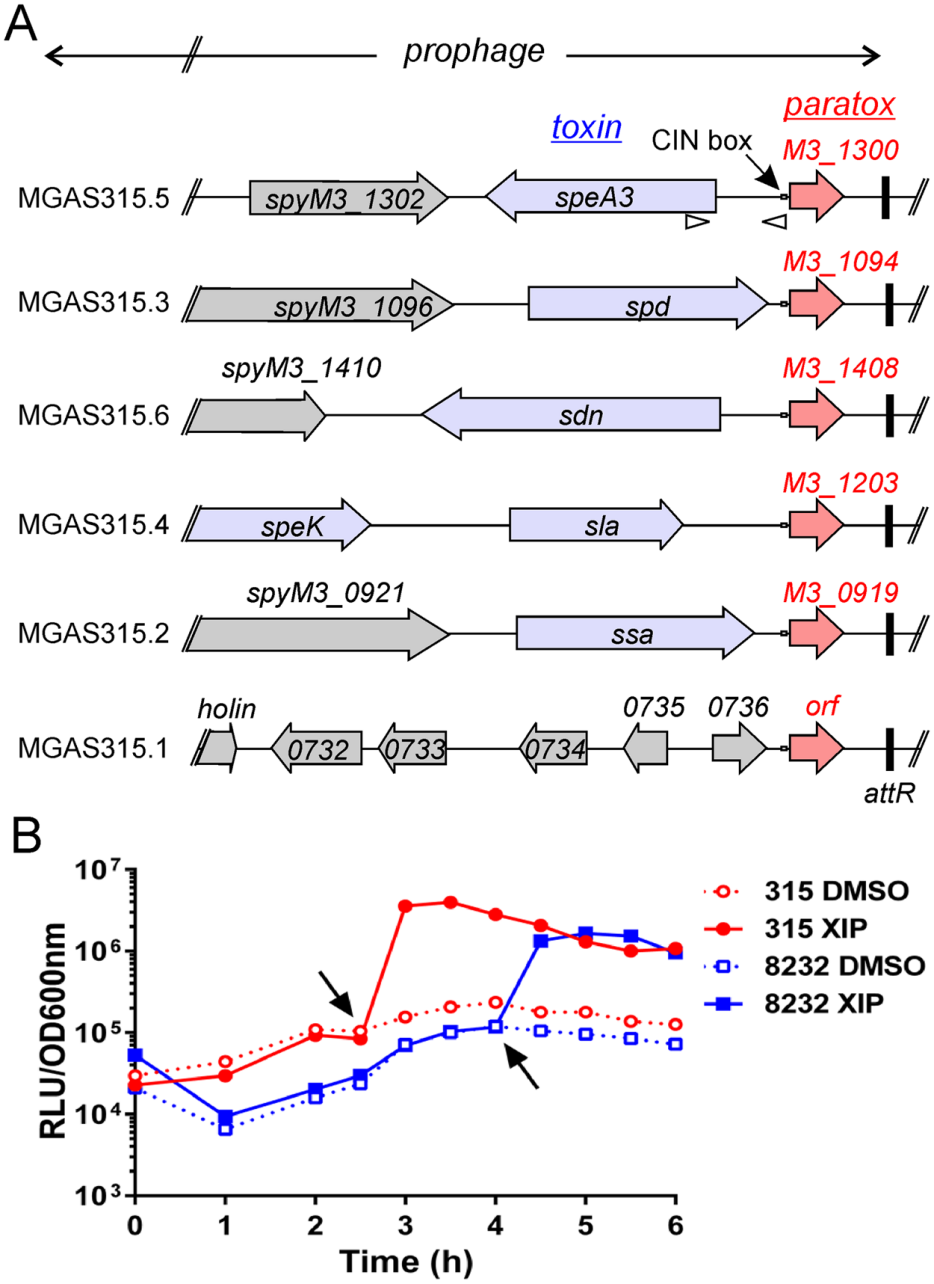
XIP induces paratox expression. (**A**) Alignment of terminal ends of the six prophage of MGAS315, indicating conservation of paratox orthologs (red) and adjacent toxin genes (blue). Black vertical bar indicates the *attR* prophage terminus. Conserved CIN-boxes (TACGAATA, marked as open rectangles) were located proximally to *prx* in every instance. Arrowheads indicate the DNA region cloned to construct the *Pprx-luxAB* reporter. (**B**) Expression of the P_*prx*_*-luxAB* reporter in the two GAS strains MGAS315 and MGAS8232 was determined in chemically defined medium with exogenous addition of XIP or a vehicle control (DMSO). Overnight cultures of GAS strains were diluted to OD_600nm_ = 0.01 and grown until early log phase (OD_600nm_ ~ 0.1), at which time synthetic XIP was added to a final concentration of 50 nM (indicated by arrows). The time of XIP addition varied due to different strain growth rates. Data shown are representative of three similar results.

Work describing the contribution of bacteriophages to the emergence and diversification of the M1T1 GAS clone revealed that each prophage contains a conserved open reading frame (ORF) of ~60aa named paratox (*prx*). *prx* is always located adjacent to a toxin within the prophage (Fig. 1A and Supplementary Fig. S1), and GAS prophages that lack a toxin also lack *prx*^28^. Moreover, *prx* was found to be at a recombination hot-spot of the prophage lysogenic conversion module and is observed to be in ‘linkage disequilibrium’ with phage-related toxins, which suggests that *prx* and the adjacent toxin are exchanged between phages as one genetic cassette. These observations led to a paratox-based recombination model for the dissemination of toxins in GAS^28^. Additionally, *prx* is not only found in GAS, it is also present in all pyogenic, bovis, and suis streptococci, which include *Streptococcus agalactiae* (Group B Streptococcus), *Streptococcus*, *dysgalactiae*, and *Streptococcus equi*.

Although *S*. *pyogenes* natural transformation has been reported^9^ and was shown to be dependent on *comRS*, the transformation frequency was relatively low compared to other naturally competent streptococci that also utilize a ComRS system (i.e. *S*. *mutans* and *Streptococcus suis*)^29,30^. In our efforts to study the regulation of natural competence in *Streptococcus spp*. by ComRS based quorum sensing, we discovered that *prx* genes contain a perfect CIN-box within their promoter region for recognition by the SigX core-RNA-polymerase complex, and that transcription of *prx* is induced by XIP. Additionally, we found that Prx binds ComR directly and inhibits activation by XIP both *in vitro* and *in vivo*. This is the first characterization of a negative regulator of ComRS quorum sensing in *S*. *pyogenes*. Additionally, an X-ray crystal structure of Prx reveals a novel protein fold with only distant structural homologues, which suggests a unique molecular mechanism of ComR-XIP inhibition. Deletion of *prx* from *S*. *pyogenes* strains failed to yield an observable phenotype in a laboratory setting related directly to natural competence, however it produced a strain of *S*. *pyogenes* over one hundred-thousand-fold more electrocompetent compared to wildtype. Interestingly, this phenotype does not appear to stem from the biochemical inhibition of ComR by Prx. Taken together we conclude that the conserved prophage protein paratox acts as an inhibitor of new DNA acquisition by pyogenic, bovis and suis streptococci.

## Results

### Paratox expression is induced by ComR and XIP

In a previous study using microarray analysis, we reported genes that were regulated by XIP in the *S*. *pyogenes* strain MGAS315^10^. We confirmed that the majority of genes induced by XIP were known genes involved in natural transformation of well-characterized model systems; however, we discovered a small subset of genes that have not been described as being a part of the competence regulon. In addition, this subset of genes included a nucleotide diphosphate kinase (*ndk*; spyM3_0584), *trmA*, and *proAB* which do not contain a CIN-Box upstream of their ORF. The microarrays used in this report^10^ were based on sequences from the *S*. *pyogenes* strain NZ131. Comparing the location of these upregulated genes in NZ131 to the MGAS315 strain, we identified that these specific genes were directly downstream of integrated prophages in MGAS315. Strikingly, copies of the same gene were found at the end of each MGAS315 prophage and were located directly upstream of *ndk*, *trmA* (SpyM3_1299), and *proB* (SpyM3_1407). Furthermore, this gene identified as *prx*, contained a perfect CIN-box upstream of the ORF, which suggested that increased transcripts of *ndk*, *trmA*, and *proAB* detected by the microarray analysis, were likely due to transcriptional read through from *prx* induction. In an evaluation of CIN-box (TACGAATA) occurrences within the MGAS315 genome, we found that 6 of 68 instances of the DNA element were located at terminal ends of prophage, within 21 nucleotides of the start codon of *prx* genes (Fig. 1A). In *S*. *pyogenes*, *prx* genes are found at the right terminal end of the majority of prophage, adjacent to phage-encoded toxins^28^ (Table 1, Supplementary Fig. S1). Due to the conserved proximity of the CIN box adjacent to the *prx* coding sequence, we tested whether production of SigX by XIP would result in the transcription of *prx* (Fig. 1B).

**Table 1:** Distribution of genetic elements and immunity within GAS strains.

Strain	M type	Prophage	paratox	ICE	CRISPR-1	CRISPR-2
SF370	1	4	1	1	7	4
MGAS5005	1	3	3	1	4	5
MGAS10270	2	5	2	2	3	4
MGAS315	3	6	6	0	1	0
SSI-1	4	6	6	0	1	0
MGAS10750	4	4	2	2	1	6
Manfredo	5	5	2	0	0	0
MGAS10394	6	8	5	0	0	0
MGAS2096	12	2	1	2	3	7
MGAS9429	12	3	1	1	3	8
MGAS8232	18	5	4	0	0	0
MGAS6180	28	4	2	3	5	2
NZ131	49	3	1	0	5	6

All sequenced *S*. *pyogenes* strains have at least one copy of paratox and one of two known allele types of *comRS*^10^. To determine if XIP influenced the transcription of paratox, luciferase reporter strains were constructed by fusion of the promoter regions of *prx* from two GAS strains to *luxAB*. The *S*. *pyogenes* strains MGAS8232 (promoter region upstream of spyM18_1444) and MGAS315 (promoter region upstream of M5005_spy1414) were chosen as they contain the two known M1 and M3 *comRS* allele types respectively^10^, and their paratox proteins are 75% identical and 88% similar. The plasmids were transferred to the respective *S*. *pyogenes* strains from which the promoters were cloned, and luciferase activity was monitored throughout growth. As shown in Fig. 1B, immediately after XIP addition at an OD_600nm_ ~0.1, *prx* was induced roughly 100-fold in both strains tested, which confirmed the CIN-box functioned as an active SigX-dependent promoter.

### Paratox functions as a negative regulator of ComR *in vitro*

**Figure 2:**
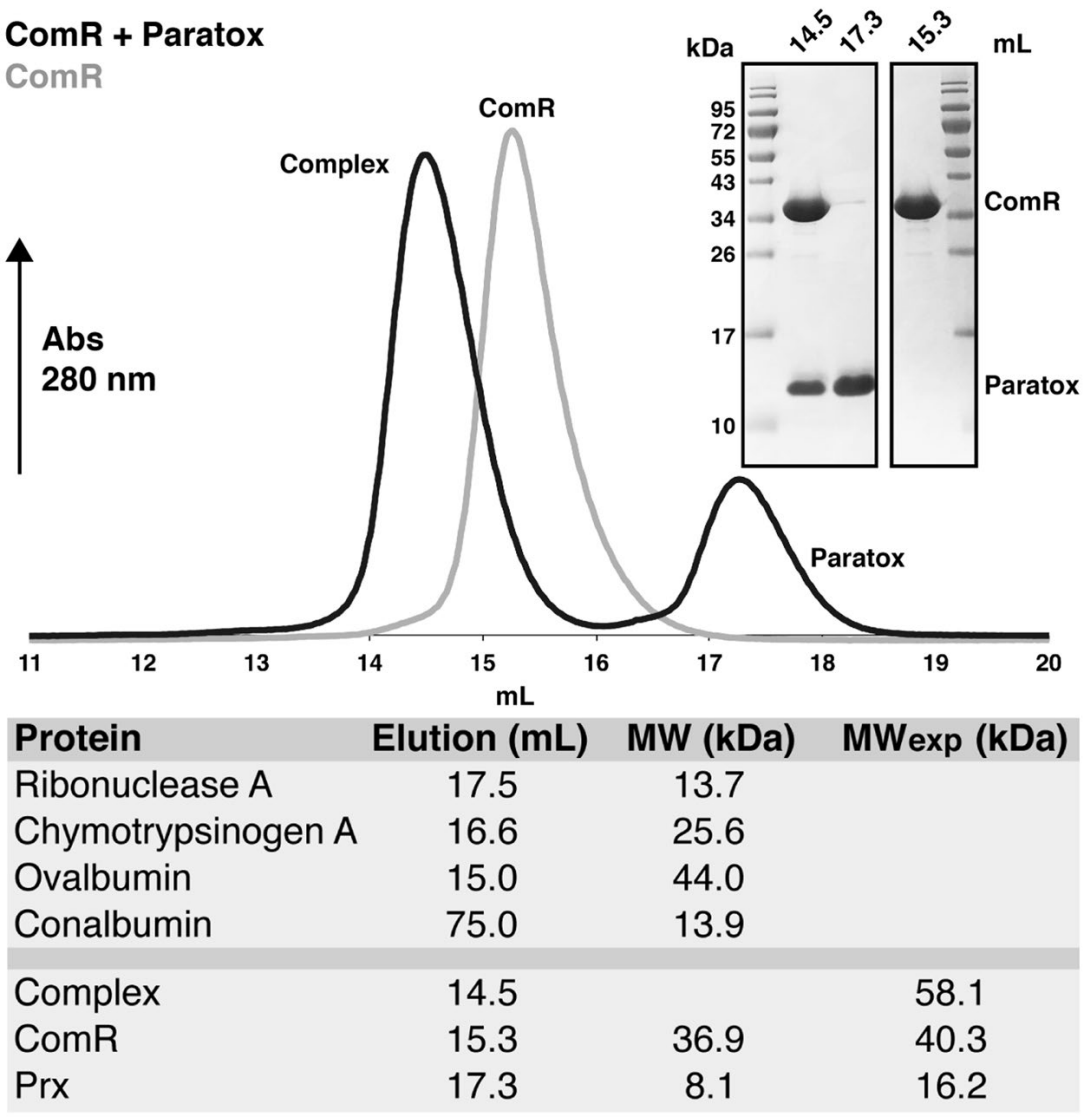
Paratox and ComR form a complex by size exclusion chromatography. Purified ComR and paratox were assayed for complex formation using an SD200 Increase 10/300 GL column (GE Healthcare). The elution profile of ComR alone (grey) and ComR + 1.5 molar ratio of paratox (black) are shown. The inset SDS-PAGE gels show the protein species present at the listed elution volumes in mL. Molecular weight standards estimate a complex of ~58 kDa or one ComR plus one or two paratox.

Given that *prx* expression was induced by XIP (Fig. 1B) we asked if Prx influenced natural competence. As natural competence can be difficult to observe for pyogenic *Streptococcus* strains in the laboratory^9,10^, we explored the possibility that recombinant GAS Prx could interact with a member of the competence regulon *in vitro*. To test this, we created recombinant forms of Prx and ComR and found that Prx forms a stable complex with ComR as determined by size-exclusion chromatography (SEC) (Fig. 2). After incubation with a molar excess of Prx, a clear shift in the elution volume of the ComR peak (grey) is observed from 15.3 mL to 14.5 mL. This represents a stable ComR-Prx complex (black) as verified by SDS-PAGE gel (Fig. 2 inset). Analysis of elution volumes using molecular weight standards shows ComR is a monomer as expected^13^ and the complex could consist of one ComR plus one or two Prx proteins (Fig. 2).

**Figure 3:**
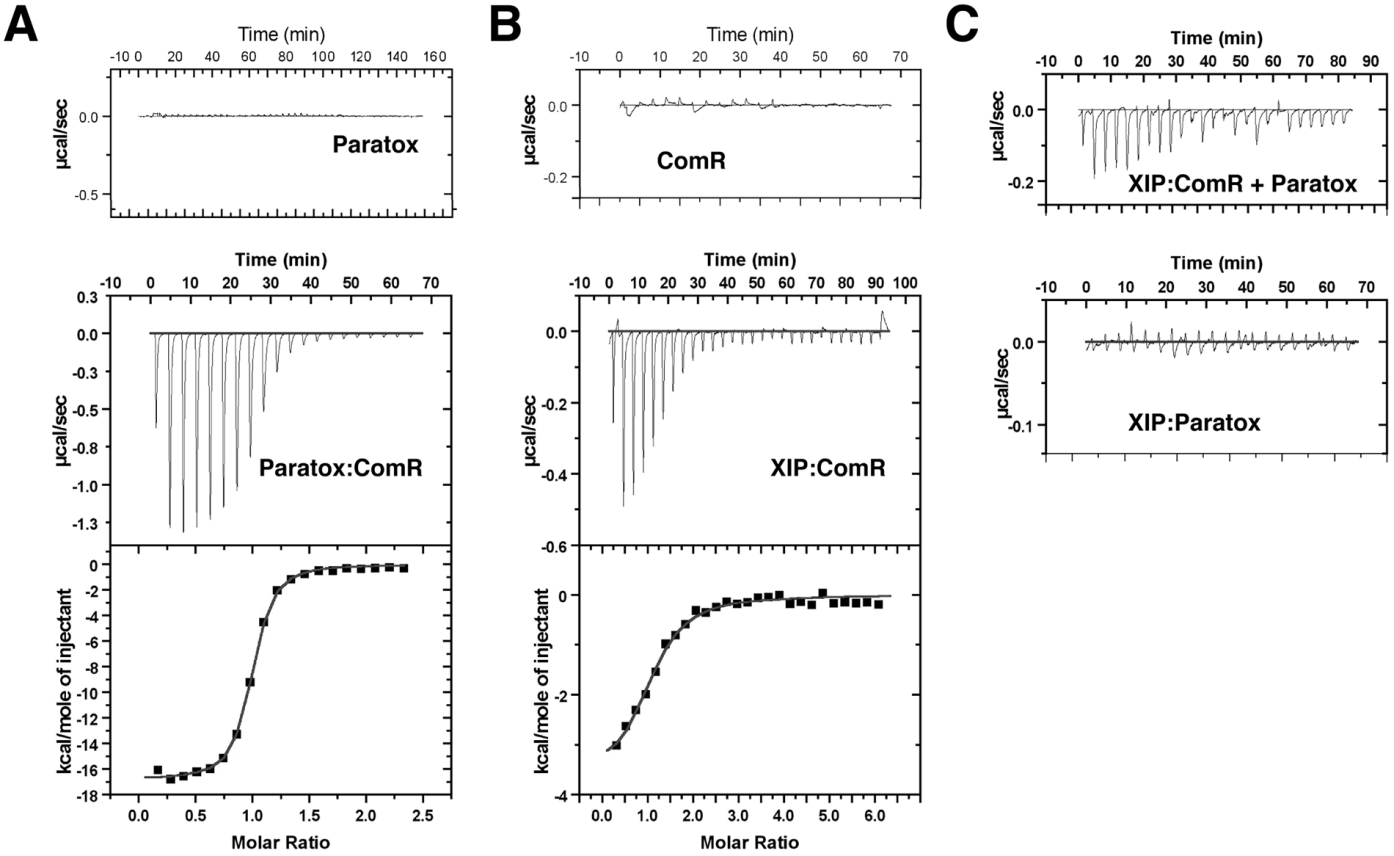
Paratox prevents binding of XIP to ComR. Isothermal titration calorimetry experiments were performed with paratox, ComR, and XIP. (**A**) Titration curves for paratox and ComR, with the control titration of paratox into buffer. N (stoichiometry) = 0.95 with a K_d_ of 0.133 μM (**B**) Titration curve for XIP and ComR, with the control titration of buffer into ComR. N (stoichiometry) = 1.01 with a K_d_ of 3.6 μM. (**C**) The top panel shows the titration of XIP into ComR + 1.5 molar ratio of paratox and the bottom panel shows the heats of titration of XIP into paratox alone. Each panel is labeled by experiment.

To further characterize the ComR-Prx interaction we assayed binding by isothermal titration calorimetry (ITC). Using a one-site model, Prx binds ComR with a K_d_ of 133 nM and a stoichiometry of 1 to 1 (Fig. 3A). Since activation of ComR, and thus natural competence, requires XIP^12,29^ we also asked if Prx has any effect on the ComR-XIP interaction. As shown in Fig. 3B, XIP binds to ComR as a 1:1 complex with a K_d_ of 3.6 μM in agreement with past results^13,14^. However, if XIP is titrated into a solution containing both ComR and Prx, the observed heats at each titration are significantly reduced (Fig. 3C top). Although Prx interferes with the binding between XIP and ComR, the binding model and reduced K_d_ were not readily apparent from the data. Moreover, as this was not due to a direct binding event between Prx and XIP (Fig. 3C bottom), Prx appears to reduce the ability of ComR to bind XIP.

**Figure 4:**
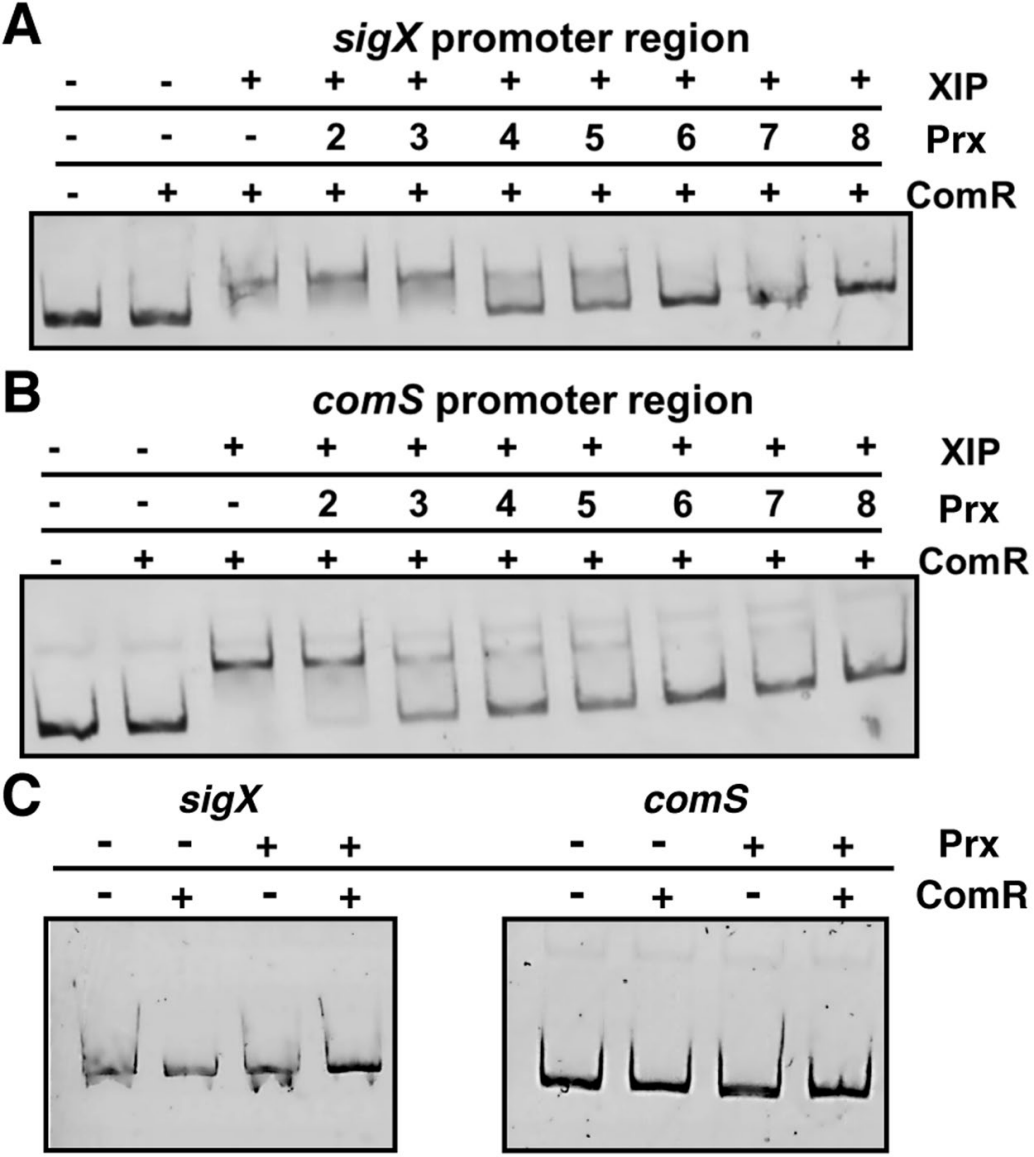
Paratox interferes with ComR-XIP DNA binding in a dose dependent manner. Electromobility shift assays using fluorescently labelled DNA probes (100 ng) comprising the *sigX* (**A**) and *comS* (**B**) promoter regions. ComR (4 μM), XIP (8 μM), and paratox (at the indicated concentrations) were incubated together at RT for 30 min. followed by the addition of DNA and allowed to incubate for an additional 15 minutes at RT. (**C**) Control experiments with paratox and ComR without XIP. The components were then run on a native PAGE gel and imaged using a Typhoon FLA 7000 phosphoimager. A DNA shift indicating a ComR-DNA interaction can be visualized only in the presence of XIP. This interaction is disrupted with the addition of paratox indicated by the loss of the DNA shift.

Although the apo-from of ComR is a monomer in solution^13^, it dimerizes and binds to DNA upon its interaction with XIP^14^. Therefore, we assessed the effect of Prx on the ability of ComR to bind DNA by electromobility shift assay (EMSA). Two promoter regions that the ComR-XIP complex binds were tested, the *sigX* promoter region^29^ and the *comS* (XIP) promoter region^12^ (Fig. 4). As expected, upon the addition of XIP to ComR there is a clear band-shift for both promoter regions, which confirmed that ComR requires XIP for activation and binding to DNA. When Prx was provided to the reaction, the amount of ComR-XIP-DNA complex is reduced in a concentration dependent manner and is no longer observed at a 2-fold molar excess of Prx (8 μM) to ComR (4 μM) (Fig. 4A,B). Additionally, this inhibition was not due to interaction of Prx with the DNA probe (Fig. 4C).

### An X-ray crystal structure of paratox reveals a novel protein fold

**Figure 5:**
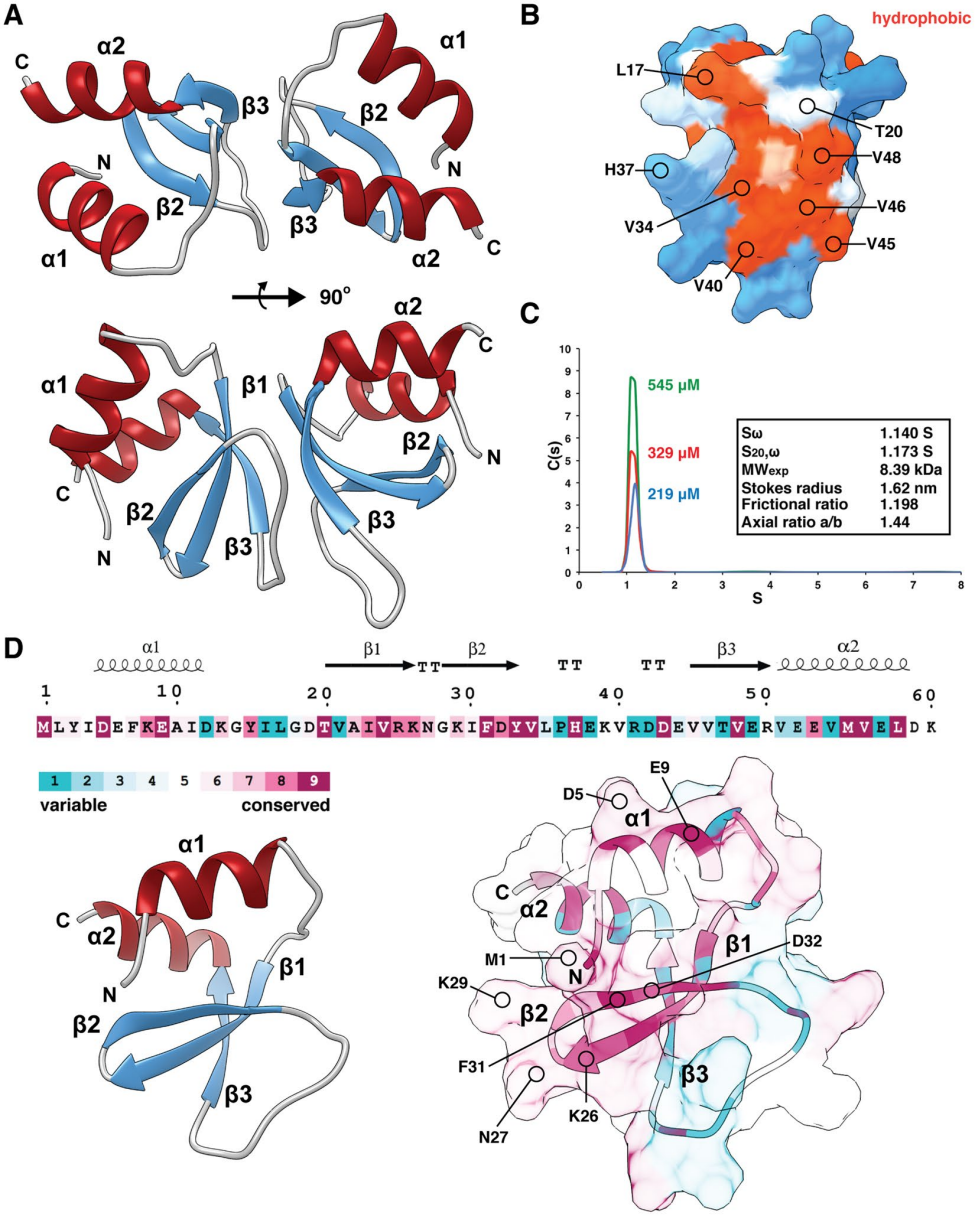
Molecular features of an X-ray crystal structure of paratox. (**A**) The paratox crystal structure contains two molecules in the asymmetric unit. Two views are shown with the interface created largely by β1, β3, and loop regions. (**B**) Analytical ultracentrifugation experiments indicate that paratox is a monomer in solution. (**C**) View of the molecular surface of one paratox monomer at the crystallographic dimer interface shows a large hydrophobic patch flanked by two conserved polar residues (T20 and H37). (**D**) Amino acid conservation of paratox as calculated by the server Consurf with secondary structure elements indicated by Espript3 (Top). The bottom left panel shows the paratox monomer and the bottom right panel the molecular surface colored by residue conservation. Residues that belong to a conserved patch on the surface of paratox are labeled. Graphics were created using the program UCSF Chimera (https://www.rbvi.ucsf.edu).

To gain further insight into the function of Prx, we pursued an X-ray crystal structure of the protein. As shown in Fig. 5A and Table 2, Prx crystallized as a dimer in the asymmetric unit. The dimer interface consists of residues from β-strands β1, β3 and the loop region between β2 and β3, which allows the N and C terminal α-helices to be positioned on the same face of the complex (Fig. 5A top). A view of the dimer interface on the surface of one monomer is shown in Fig. 5B with several of the interacting residues indicated. These residues are homologous between several annotated Prx sequences (Supplementary Fig. S2A) and create a large hydrophobic patch at the interface with two channels large enough to accommodate the binding of a small molecule ligand (Supplementary Fig. S2B). As calculated by MOLEonline/2.5^31^, the pore or cavity sizes are 380Å^3^ and 993Å^3^. Although analysis by SEC indicates a possible dimer (Fig. 2), analysis with PISA^32^ gives a complex significance score of 0.1, which suggests a high probability that dimerization is a crystal packing artifact. To reconcile the Prx oligomerization state, we performed sedimentation velocity experiments with increasing Prx concentrations (Fig. 5C). As demonstrated by analytical ultracentrifugation (AUC), Prx is a monomer (8.39 kDa) in the buffer conditions used in our biochemical experiments. Additionally, the fitted sedimentation data calculate a stokes radius and axial ratio indicative of a globular protein in agreement with the observed Prx monomer (Fig. 5D left).

**Table 2:** X-ray crystallographic data collection and refinement statistics.

Data Collection	
Space group	P22_1_2_1_
Cell dimensions	
a, b, c (Å)	26.69, 56.82, 88.37
α, β, γ (°)	90.0, 90.0, 90.0
Wavelength	0.979408
Resolution (Å)	50.0–1.56
R_meas_	11.4 (61.6)
CC(1/2)	99.3 (98.5)
*I/σI*	14.1 (4.5)
Completeness (%)	99.3 (98.2)
Redundancy	11.7(11.8)
Refinement	
Resolution (Å)	47.79–1.56
Number of monomers in asymmetric unit	2
R_work_/R_free_	16.2/18.4
No. atoms	
Protein	2499
Water	200
*B*-factors	
Protein (Chain A, B)	27.6/32.6
Water	32.9
R.m.s deviations	
Bond lengths (Å)	0.008
Bond angles (°)	0.885
Ramachandran plot (%)	
Favored	98
Allowed	2
Outliers	0

The properties of the molecular surface of the Prx monomer were explored using the Consurf server^33^ to plot residue conservation (Fig. 5D right) and the Adaptive Poisson-Boltzman Solver^34^ to calculate electrostatic potential (Supplementary Fig. S2C). Both β1 and β2 appear to be highly conserved, and along with the N-terminus form a surface of mostly polar and charged residues (Fig. 5D). A surface composed of one face of α1 (D5, L8, and E9) and α2 (M55, V46) also cluster into a conserved patch, and both surfaces tend toward an electronegative electrostatic potential (Supplementary Fig. S2C). Given that the surface of ComR is also highly electronegative^13^ it is unclear which, if either conserved surface is the likely site of interaction with ComR.

Structural homologues to Prx were searched for using both the PDBeFold^35^ and the Daliserver^36^. Each algorithm returned only a couple dozen hits with a highest Q-value of 0.24 (PDBeFold) and Z-scores 3.0 or below (Dali server), which indicates that Prx only has distant structural relatives. Structural alignments with four of the representative protein families are shown in Supplementary Fig. S3.

The observed Prx dimer appears superficially similar to a Bateman fold^37^, with each Prx monomer as the individual CBS (cystathionine β-synthase) domain^37^ as shown in an alignment with human AMP-activated kinase^38^ (Supplementary Fig. S3A). CBS domains are similar in size to Prx (60 residues) and function as regulatory subunits by binding small molecule ligands (AMP, NAD) at the cleft between the CBS domains. As Prx may regulate ComR (Figs 3, 4 and 5) and the dimer cleft could accommodate a ligand (Supplementary Fig. S2), this comparison is attractive. However, unlike Prx the individual CBS domains are covalently linked and typically tethered to the enzyme they regulate^37^. The closest structural homologues as determined by PDBeFold (Q = 0.24, 9% sequence identity) are MbtH-like proteins (MLPs) (Supplementary Fig. S3B). MLPs are small proteins that bind and enhance the activity of nonribosomal peptide synthases (NRPSs), which in bacteria are involved in the production of siderophores^39^. The closest match found by the Dali server (Z-score 3.0, 5% sequence identity) is the Rad9A/Rad1/Hus1 (human) DNA damage checkpoint complex^40^ where Prx can be aligned to one of the repeating units of the ring-like oligomer (Supplementary Fig. S3C). Additionally, the protein GpW of bacteriophage λ^41^ appears to be a distant relative of Prx (Q = 0.17, 18% sequence identity) (Supplementary Fig. S3D). GpW is a component of the virus particle joining the head and tail, and is thought to participate in protein-protein and protein-DNA interactions^42^.

Although these protein folds have regulatory and protein-protein binding activities like Prx, their distant structural relation further highlights that Prx is a newly characterized protein fold. Furthermore, the lack of similarity prevents their use as a guide for rational choices in site-directed mutagenesis studies. This combined with multiple conserved surfaces on Prx (Fig. 5D) indicate that future structural studies, such as a co-crystal complex, will be required to accurately study the ComR-Prx binding interface.

### Paratox serves as a repressor of the competence regulon *in vivo*

**Figure 6:**
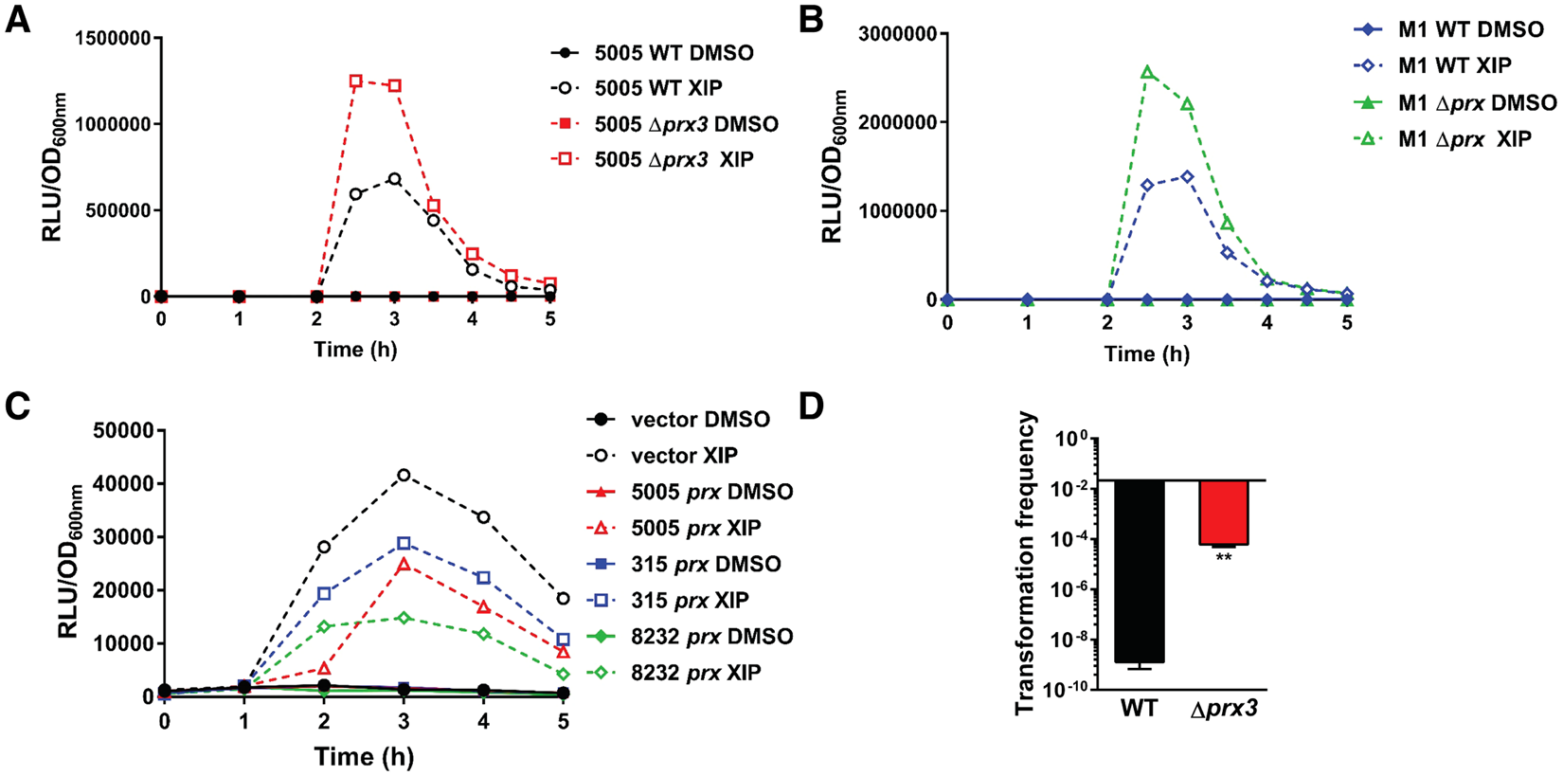
Paratox represses *sigX* and late competence gene expression and is involved in GAS electrocompetence. Expression of the P_*ssb*_*-luxAB* reporter in MGAS5005 wildtype, MGAS5005 Δ*prx3* (**A**), M1 wildtype, and M1 Δ*prx* (**B**) was determined in chemically defined medium with exogenous addition of XIP or a vehicle control (DMSO). Overnight cultures of GAS strains were diluted to OD_600nm_ = 0.05 and grown until early log phase (OD_600nm_ ~0.1), at which time synthetic XIP was added to a final concentration of 50 nM. Data shown are representative of three similar results. (**C**) Expression of the integrated P_*sigX*_*-luxAB* reporter in *S*. *mutans* that contained vector only, MGAS5005 *prx3*, MAGS315 *prx*, or MGAS8232 *prx in trans* was determined in chemically defined medium with exogenous addition of XIP or a vehicle control (DMSO). Overnight cultures of *S*. *mutans* strains were diluted to OD_600nm_ = 0.01 and grown until early log phase (OD_600nm_ ~0.1), at which time synthetic XIP was added to a final concentration of 200 nM. Data shown are representative of three similar results. (**D**) Wildtype MGAS5005 and the Δ*prx3* deletion strain were assayed for transformation efficiency by electrocompetence. The transformation frequency was determined by comparing the amount of transformants vs. the control cultures. Error bars represent the standard error mean (SEM). Asterisks indicate the statistical significance (**P < 0.006).

Using an array of biochemical methods, we have shown that ComR and paratox interact directly and that this interaction prevents the binding of ComR to its DNA target *in vitro*. To determine the impact paratox has on ComR-dependent regulation of the competence regulon *in vivo*, we generated a *prx* deletion strain in MGAS5005 of prophage MGAS5005.3, which contains the toxin streptodornase *sdaD2*; this strain is referred to as Δ*prx3*. This strain was chosen as it contains a minimal amount of *prx* alleles (3) compared to other strains that are comprised of up to 6 copies (Table 1). We also created a separate paratox deletion strain in M1 SF370, which comprises a single unannotated paratox gene located just upstream of *mf3* at the end of the phage element (Fig. 1); this strain is referred to as M1 Δ*prx*. A luciferase reporter that was previously constructed to measure the transcription of the XIP-induced late competence gene *ssb* (single stranded binding protein)^10^, was transferred into MGAS5005 and M1 SF370 wildtype. Likewise, this reporter was also transferred into paratox deletion strains MGAS5005 Δ*prx3* and M1 Δ*prx*. As shown in Fig. 6A,B, after the exogenous addition of XIP expression of *ssb* was further increased in both *Δprx* strains compared to their respective wildtype strains. This supports our *in vitro* data which illustrates that Prx negatively influences the competence pathway via interaction with ComR.

To further examine the outcome of Prx on ComR activity *in vivo*, we tested the effect of paratox in *S*. *mutans*, a species that requires ComR and XIP for natural competence but does not contain paratox genes. To do this, plasmids that contained three different GAS *prx* genes under their native CIN-box-containing promoters from strains MGAS5005, MGAS315, and MGAS8232 were constructed and separately introduced into an *S*. *mutans* strain that harbors a *sigX* transcriptional reporter integrated into the chromosome^43,44^. The empty vector pJC156 without paratox was used as a control. As expected, when XIP was added to the reporter strain that comprised the empty vector a 40-fold increase in *sigX* transcription was observed. In contrast, the strains that contained the paratox genes had ~2-fold or greater reduction in *sigX* expression, which signified that the paratox proteins suppressed the ability of ComR to activate *sigX* to its full capacity. Taken together these data demonstrate that Prx negatively influences the ComR-dependent competence regulon.

### A single paratox deletion mutant increases electroporation-mediated transformation in *Streptococcus*

Given that paratox directly interacts with ComR and interferes with ComR–XIP–DNA complexes both *in vitro* (Figs 2, 3 and 4) and *in vivo* (Fig. 6), we hypothesized that the deleted paratox strains may show a phenotype of increased natural transformation. Both wildtype and Δ*prx* strains yielded no transformants when grown planktonically with the addition of XIP as previously described for measuring natural competence^10^. We did discover unexpectedly that when DNA was introduced via electroporation, the MGAS5005 Δ*prx3* mutant strain generated >100,000 fold increase in transformants compared to the wildtype (Fig. 6D). In contrast, the M1 Δ*prx* strain (which has a single *prx* allele) exhibited electrocompetence similar to the M1 wildtype. It is currently unknown if deletion of other *prx* alleles will have a similar effect in MGAS5005, or what the phenotype of *prx* deletions are in other strains. However, we confirmed that this surprising result in MGAS5005 was not due to the loss of DNase activity as the toxin downstream of paratox encodes *sdaD2*, a streptodornase. Expression of *sdaD2* was confirmed in the Δ*prx3* strain by semi-quantitative RT-PCR (data not shown). These results suggested that the electrocompetence phenotype exhibited by *prx3* is not attributed to the loss of DNase activity, but likely some other unknown mechanism.

## Discussion

In our work described here, we reveal that the conserved GAS prophage gene *prx* is activated by the ComRS quorum sensing system and inhibits the activation of ComR both *in vitro* and *in vivo*. Additionally, a comparison of an X-ray crystal structure of Prx with known protein structures reveals that it adopts a novel protein fold, which suggests a unique molecular interaction with ComR. Finally, creation of a *Δprx* strain had the unpredicted effect of increased electrocompetence of MGAS5005 by over 100,000 fold.

These findings indicate that paratox may serve as a negative-feedback mechanism upon the ComRS sensory system. Interference with ComR would presumably inhibit or limit expression of the alternative RNA polymerase sigma factor SigX, and hence, inhibit induction of the competent state. Although evidence for natural transformation under laboratory conditions for *S*. *pyogenes* and other pyogenic streptococci is scarce, even when the ComRS–SigX pathway is induced^10^, specialized conditions, such as biofilm growth on epithelial surfaces, have provided evidence that natural transformation remains a functional attribute^9^. The high frequency of CIN box-associated paratox genes present on *S*. *pyogenes* and other pyogenic, bovis, and suis group prophages strongly suggests there exists a benefit to the prophage to have acquired the ability to express *prx* upon ComR induction. Paratox was identified as a conserved location at terminal ends of prophage where homologous recombination occurs to generate phage diversity^28^. As demonstrated here that paratox binds and inhibits ComR, it can be inferred that activation of *prx* at the very time when competence initiates provides an efficient and precise means for phage to block development. Events concurrent with competence induction, which include DNA integration and repair, are destabilizing to prophage^45–48^. Thus, blocking uptake and integration of DNA could serve as a self-preservation mechanism for phage.

Prx is a novel protein fold with multiple conserved surfaces (Fig. 5 and Supplementary Fig. S3), which makes clues to the mode of interaction with ComR not readily discernable. Furthermore, as ComR undergoes a large conformational change upon activation by XIP^14^ it is not clear which surface on ComR is a likely interaction site. Our previous work has shown that the C-terminal TPR (tetratricopeptide repeat) domain of ComR creates a binding site with a conserved and variable residue face for the selection of an XIP pheromone^13^. Additionally, in the apo-conformation ComR is a monomer with the N-terminal DNA binding domain (DBD) held tightly to a conserved patch on the TPR domain to shield DNA interacting residues from the solvent^13^. Interaction with XIP alters the TPR so as to release the DBD for interaction with DNA and dimerization that includes a domain swap of the DBD^14^. Although our data shows that Prx has a binding affinity for ComR higher than XIP (133 nM vs 3.6 μM) and in ITC experiments Prx interferes with XIP binding (Fig. 3), we cannot currently conclude that Prx binds in or over the XIP pocket. Equally possible is that Prx binds ComR at another surface so as to stabilize the apo-conformation and prevent a conformational change. This hypothesis is supported by our observations that ComR can bind non-activating XIP molecules at lower affinity^13^ and the reduced heats present in the ComR-Prx-XIP ITC experiment (Fig. 3). EMSA data indicate that the presence of Prx prevents ComR from binding DNA in an XIP dependent manner, and Prx does not directly bind the DNA probe (Fig. 4C). However, given the known structural changes for ComR activation^13,14^ and that Prx inhibits the binding of XIP (Fig. 3) it is unlikely that Prx simply binds the ComR DBD to shield it from interaction with DNA. Together our data suggest that Prx stabilizes the apo-ComR conformation by binding at the XIP pocket or other TPR surface to potentially trap it in an inactive state.

It is also important to note that our *in vitro* binding experiments used Prx from *S*. *pyogenes* and ComR from *S*. *mutans*. We were unable to produce recombinantly expressed ComR from MGAS315 or MGAS5005 at both sufficient quantity and purity for our experiments. Although *S*. *mutans* rarely harbors bacteriophage, and thus does not have an identified Prx homolog, it does have a type-II ComR similar the Pyogenic group with XIP having a conserved double tryptophan motif^29^. This immediately suggests that Prx recognizes a structural feature or surface common to type-II ComR proteins such as the conserved face of the XIP binding pocket^13^. Moreover, the question of whether Prx can recognize and inhibit type-I (*S*. *salivarius*)^49^ and type-III (*S*. *suis*) ComR proteins^13^, or if it has evolved to specifically inhibit the quorum sensing receptor of its specific group remains to be answered. This is an especially pertinent question as our research has shown that the sequences of both ComR and its XIP are variable and correlate to the ability of different *Streptococcus* species to cross-talk and recognize other species’ peptide pheromones^13^. Given that *Streptococcus* can discriminate between XIP signals^13^, Prx inhibits activation of the natural competence receptor, and that Prx proteins are genetically linked to a specific toxin^28^; is there a pattern of horizontal gene transfer between *Streptococcus* species that is dependent upon a balance between XIP recognition by ComR and Prx inhibition of ComR? Furthermore, is this a balancing act that is controlled by the prophage to inhibit natural competence when it needs to favor transduction, such during conversion to a lytic or pseudolysogenic state^50^? As the toxin linked to Prx is often an extracellular DNase, is the role of Prx to assure that any of the partially digested DNA from the environment is not used in recombination into the bacterial or phage genome? Additional research will be required to answer these questions.

Although the molecular mechanism of ComR inhibition by paratox remains to be determined, a clue may lay with the observed crystal packing of the Prx dimer. It is common for proteins in their monomeric state to use their natural oligomeric binding surfaces as crystal packing contacts. For example, we observed this with monomeric ComR^13^ and it was observed for the Gram-positive secretion chaperone PrsA in some species^51,52^. Given the distant relation to CBS domains and potential ligand binding pockets (Supplementary Figs S2 and S3) it is possible that Prx dimerization is regulated by a small molecule. It is tempting to speculate that this regulation is keyed to the physiological conditions that are needed to observe natural competence for *S*. *pyogenes*. Although an enticing hypothesis, screening for a potential Prx ligand and a stable Prx dimer for biochemical studies are required to test such a model, in addition to further in-depth genetic analysis using multiple *prx* deletion strains.

To correlate our biochemical data directly to an *in vivo* activity, we created paratox deletions in two different GAS strains, MGAS5005 that harbors 3 paratox genes, and M1 SF370 which contains a single paratox gene. The paratox deletion strains did exhibit enhanced late competence gene induction via the *ssb* transcriptional reporter, which rapid decline was most likely due to *sigX* degradation by the ClpP protease^10,53^. Interestingly, we saw greater differences between the WT and the M1 paratox mutant compared to MGAS5005. We hypothesize that this observation was due to the presence of 2 other paratox genes in MGAS5005 that could readily bind ComR and prevent downstream induction events, whereas the M1 strain harbors a single paratox gene that was removed. In addition, we measured the induction of *sigX* in *S*. *mutans*, a species whose natural competence requires ComR and XIP, and one that does not contain any paratox genes. By separately providing three different GAS paratox genes to this species *in trans*, we revealed that *sigX* expression was notably less than when paratox was absent. Together our *in vitro* and *in vivo* data reveal that paratox represses the ability of ComR to activate the competence regulon via direct binding to ComR, and thus acts to inhibit ComRS quorum sensing in *S*. *pyogenes*.

As Prx inhibits ComR *in vitro* (Figs 3 and 4) and negatively influences downstream ComR-mediated events *in vivo* (Fig. 6), why did we not observe an increase in natural competence? As noted previously, natural competence for *S*. *pyogenes* remains extremely difficult to observe in a laboratory setting. This suggests that the simplest answer is that even with the deletion of *prx* our experimental system does not meet the required conditions for natural competence for *S*. *pyogenes*. Furthermore, as many species of *Streptococcus* have multiple *prx* alleles (Table 1) they could act redundantly requiring the deletion of all copies of *prx*. However, as our *prx* deletion mutants did not show an increase in natural competence, but do actively repress XIP dependent expression of the competence regulon (Fig. 6), this indicates that other factors likely play a role in the inability of GAS to be naturally competent

Most GAS strains, which include MGAS5005 are difficult to manipulate genetically^54^. This observation was confirmed in this study; however, the removal of a single copy of *prx* in MGAS5005 resulted in a 100,000-fold increase in electrotransformation efficiency. Although the mechanism for this result is not understood, there are two possible means; Prx is physically blocking DNA from entering the cell or Prx is manipulating the DNA once it enters the cytoplasm either directly or by an indirect mechanism. This is currently under investigation. Coincidently, strains with higher copies of paratox such as MGAS5005 and MGAS315 are difficult to genetically manipulate^54^, whereas the strain NZ131 has only one copy of *prx* and a mutated *comR*^10^ and is highly electrocompetent^55,56^. Nonetheless, this is the first report to show that a quorum sensing-controlled phage protein affects electrocompetence, and this could provide a useful genetic tool for *Streptococcus*. In contrast to the electrocompetence frequency we observed with MGAS5005 Δ*prx3*, we found that the electrotransformation efficiency in M1 Δ*prx* was similar to the M1 wildtype. This suggests that other paratox alleles (e.g. M1 paratox) may have different binding partners. Moreover, this hypothesis is further validated as paratox alleles are not 100% identical (most are >75% identical). In fact, to date we have identified at least 13 paratox genes that differ at the amino acid level amongst GAS strains.

At the genetic level, *prx* genes are linked to a specific phage toxin and are located at recombination hot-spots^28^. In the context of our finding that Prx acts to inhibit natural competence, it is tempting to speculate that the biological role of Prx is to protect the genetic stability of the phage. Specifically, the ability to prevent *S*. *pyogenes* from acquiring new DNA not only safeguards the prophage genome and but also allows the phage to steer the evolution of its host. Furthermore, Prx might also allow the prophage to preferentially, or selfishly, propagate a *prx* linked toxin gene. Additional experimentation is required to pursue these hypotheses, notably in both phage genetics and transduction in *Streptococcus*.

Although many questions remain about the biology of paratox and its relation to both natural competence and the phage life-cycle, our research has provided the ground work for another avenue of study into the host-phage relationship. Specifically, we have detailed the discovery of a previously unknown and interesting intersection between horizontal gene transfer by transduction and by natural competence in *Streptococcus*.

## Methods

### Bacterial strains, media, and plasmids

Bacterial strains, plasmids, and oligos used in this study are listed in Table S1. Cultures of *S*. *pyogenes* and *S*. *mutans* were grown in closed tubes at 37 °C in a chemically defined medium (CDM)^10,29,57^ or Todd Hewett Broth plus 0.2% yeast extract (THY). For selection, cultures were plated on THY agar (1.5%) with the appropriate antibiotic. *Escherichia coli* was routinely grown in lysogeny broth (LB). Selective levels for antibiotics for *S*. *pyogenes* and *S*. *mutans* were 1.5 μg/ml erythromycin, 4 μg/ml chloramphenicol, 500 μg/ml kanamycin, and 200 μg/ml spectinomycin. For *E*. *coli*, the antibiotics used were 100 μg/ml ampicillin, 500 μg/ml erythromycin, 50 μg/ml kanamycin, and 10 μg/ml chloramphenicol. The paratox mutant strains in MGAS5005 (MW393) and SF370 (MW405) were created via allelic replacement with a kanamycin or chloramphenicol resistance cassette respectively. To construct the MGAS5005 and M1 SF370 paratox mutants (MW393 and MW405), upstream and downstream regions (1100-bp each) from spyM3_1300 and the M1 paratox ORF were amplified using oligos 5005 prx3 US F/5005 prx3 US R, 5005 prx3 DS F/5005 prx3 DS R, M1 prx US F/M1 prx US R, and M1 prx DS F/M1 prx DS R. The kanamycin resistance cassette was amplified from pOsKaR^54^ using primers Kan F/Kan R and the chloramphenicol cassette was amplified from pJC156 (ref) using primers Cat F/Cat R. These amplified products were cloned separately into pFED760^10^ using their respective restriction sites to create the MGAS5005 *prx3* knockout construct pWAR349 and the M1 *prx* knockout construct pWAR377. The plasmids were cloned, transformed in GAS and created as described previously^10^. To introduce paratox genes into *S*. *mutans*, the promoter regions and paratox ORFs were amplified from MGAS5005, MGAS315, and MGAS8232 genomic DNA using oligos 5005 prx F/R, 315 prx F/R, and 8232 prx F/R and cloned separately into pJC156. The resulting plasmids pWAR362, pWAR322, and pWAR323 were then transformed into the *S*. *mutans* strain, MW30 which harbors an integrated P_*sigX*_-*luxAB* within a neutral site of the chromosome as described previously^43,44^.

### Transcriptional fusion assays

The MGAS315 *prx* (spyM3_1300) transcriptional fusion was constructed by amplifying the *prx* promoter region using the oligos 315 Pprx F/ R with MGAS315 genomic DNA as template. The resulting 300-bp product was inserted into pWAR303 using the SalI and PstI sites to create pWAR285. The MGAS8232 *prx* (spyM18_1444) transcriptional fusion was constructed by amplifying the *prx* promoter region using the oligos 8232 Pprx F/R with MGAS8232 genomic DNA as template. The resulting 300-bp product was inserted into pWAR303 using the SalI and PstI sites to create pWAR286. Both transcriptional fusions pWAR285 and pWAR286 were transformed into MGAS315 and MGAS8232 respectively as previously described^10^. The transcriptional fusion pWAR205^10^ was transformed into MGAS5005, M1 SF370, MW393, and MW405 separately. For GAS reporter assays, strains MW332, MW333, MW406, MW407 were grown in CDM as described previously^10^ using 50 nM of M1 and M3 XIP as indicated in the figure legends. For *S*. *mutans* reporter assays, strains MW48, MW49. MW50, and MW51 were grown in CDM as described using 200 nM *S*. *mutans* XIP^29^.

### Protein expression and purification

The MGAS315 paratox expression vector was constructed by amplifying the ORF of spyM3_1300 using oligos 315 prx pET F/315 prx pET R using MGAS315 genomic DNA as template. The resulting product was inserted into pET-21a using NdeI and XhoI sites to create pWAR331. The expression vector was then transformed into the *E*. *coli* strainC41(DE3). For paratox purification, cells were grown in LB media at 37 °C until an OD of >0.6 at 600 nm. Protein expression was induced by the addition of 1 mM isopropyl β-D-1-thiogalactopyranoside (IPTG). The temperature was reduced to 20 °C and cultures were allowed to grow overnight. Cells were harvested by centrifugation and resuspended in lysis buffer (50 mM Tris pH 7.5, 500 mM NaCl, 25 mM imidazole) for lysis with an Emulsiflex-C5 (Avestin). Lysate was cleared by centrifugation at 16,000 rpm for 30 minutes and the supernatant passed over a nickel NTA gravity column (Pierce). The column was washed with 50 column volumes of chilled lysis buffer. Protein was eluted with 5 column volumes elution buffer (50 mM Tris pH 7.5, 500 mM NaCl, 500 mM imidazole) and further purified using an SD75 16/60 superdex gel filtration column (GE Healthcare) via AKTA (GE Healthcare) at 4 °C. The final buffer for paratox was of 20 mM Tris pH 7.0, 100 mM NaCl, 0.5 mM betamercaptoethanol (β-ME). Selenomethionine labeled protein was produced using metabolic inhibition^58^. Briefly, cells were grown in M9 media at 37 °C followed by reducing the temperature to 20 °C and adding 0.05 g/L seleomethionine, leucine, valine, proline, and 0.1 g/L lysine, threonine, phenylalanine. The cells were allowed to grow for an additional 30 minutes before induction with 1 mM IPTG.

To construct the *S*. *mutans* ComR (UA159) expression vector, *comR* was amplified using oligos UA159 comR pET F/ UA159 comR pET R and *S*. *mutans* UA159 genomic DNA as template. The resulting product was inserted into pET-15b using NdeI and XhoI sites to create pWAR368. The expression vector was then transformed into the *E*. *coli* strain BL21(DE3). For ComR purification, induction, lysis, and affinity chromatography were performed as paratox. The buffer used in gel-filtration for *S*. *mutans* ComR was 20 mM Tris pH 7.5 100 mM NaCl 1 mM β-ME.

### Size exclusion chromatography

Purified paratox and ComR were incubated together at 4 °C for 10 minutes at a ratio of ~3 to 1 then injected onto an SD200 Increase 10/300 GL column (GE Healthcare). The column buffer used was 20 mM Tris pH 7.5 100 mM NaCl 1 mM β-ME. Molecular weights were estimated using the molecular weight standard kits (GE Healthcare).

### Isothermal titration calorimetry

Paratox and ComR were dialyzed overnight at 4 °C into the same buffer stock of 20 mM Tris pH 7.5 100 mM NaCl 1 mM β-ME. paratox at a concentration of 240 μM was injected into dilute ComR at 20 μM.

HPLC purified synthetic peptide XIP for *S*. *mutans* U159 (GLDWWSL) was obtained from NeoScientific (Woburn, MA). XIP was reconstituted DMSO and diluted into the experimental buffer followed by centrifugation at 12,000 rpm to clear undissolved or precipitating material. A matching DMSO concentration was maintained in all ITC buffers. XIP was used at 600 μM concentration and injected into 20 μM ComR. To test XIP binding to ComR in the presence of paratox, ComR was incubated with a 1.5 molar excess of paratox for 10 minutes prior to injection of XIP. All experiments were performed using a VP-ITC calorimeter (GE Healthcare) at 25 °C. Controls included titration paratox into buffer, XIP into buffer, XIP into dilute paratox, and the titration of buffer into ComR. The final heats of binding were analyzed using Origin Software (GE Healthcare) using a one-site model.

### Electromobility shift assays

Fluorescently labeled DNA probes were constructed by amplifying the promoter regions of *sigX* and *comS* using the oligos UA159 PsigX F/FAM UA159 PsigX R and UA159 PcomS F/FAM UA159 PcomS R respectively. ComR (4 μM), XIP (8 μM), and paratox at the indicated concentrations were incubated together in a reaction as previously described^59^ for 30 min. at room temperature, followed by the addition of the DNA probe (100 ng) and incubated for an additional 15 min. at room temperature. The reactions were then applied to a 5% native PAGE gel and imaged on a Typhoon FLA 7000 Phospimager.

### Protein crystallization

Paratox MGAS315 was concentrated to 13 mg/mL for initial screening in commercially available conditions with a Tecan Freedom Evo 200 robot at the University of Illinois at Chicago Research Resources Center High Through-put screening facility. The crystallization conditions were 13 to 26 mg/mL paratox with a 1:1 mixture of 30% PEG 4000 0.1 M Ammonium acetate and 0.1 M sodium citrate pH 5.6. Crystals were grown by sitting drop vapor diffusion at 4 °C.

### Data collection and refinement

Diffraction data was collected at the Advanced Photon Source at Argonne National Laboratories as part of the LS-CAT, Sector 21. Protein crystals were prepared by cryoprotection in mother liquor with 35% PEG4000 and flash freezing in liquid nitrogen. Data was processed using XDS^60^ and phases determined by single anomalous diffraction (SAD) on data collected near the selenium peak using both the Phenix package^61^, Crank2^62^ and SHELX^63^. The initial model was further built and refined using Coot^64^, Refmac5^65^ from the CCP4 suite of programs^66^, Phenix^61^, and TLS refinement^67^. The final model has an R/Rfree of 16.2/18.3% with 100% of residues in the allowed region of the Ramachandran plot. The coordinates and structure factors (code 6CKA) have been deposited to the Protein Data Bank, Research Collaboratory for Structural bioinformatics, Rutgers University, New Brunswick, NJ (www.rcsb.org). Molecular graphics and analysis were performed with UCSF Chimera^68^.

### Analytical ultracentrifugation

Paratox was buffer exchanged into 20 mM Tris pH 7.5 100 mM NaCl 1 mM β-ME by gel-filtration using an SD75 16/600 column (GE Healthcare) then prepared as three samples at 219 μM, 329 μM, and 545 μM for velocity sedimentation experiments. The samples and buffer blanks were loaded into an AN-60Ti with double-channel centerpieces (Beckman Coulter). Data was collected using a ProteomeLab XL-1 (Beckman Coulter) at 50,000 rpm and 20 °C with 1 scan per minute at 280 nm in absorbance mode. A total of 370 scans were collected for each sample. Data was analyzed using SEDFIT and a continuous distribution model^69^. For data fitting the buffer density and viscosity were calculated using SENTERP to be 1.00293 and 0.0169 poise respectively, and vbar for paratox to be 0.744 mL/g.

### Electrocompetence assays

MGAS5005 wildtype, MGAS5005 Δ*prx3*, M1 SF370 wildtype, and M1 ∆*prx* were grown to mid log phase in THY that contained 20 mM glycine. Hyaluronidase was added immediately before the cells were pelleted by centrifugation. Cells were then washed for a total of two times with 15% glycerol and concentrated 250-fold. 50 μl aliquots were used for electroporation where no DNA or 2 μg of plasmid DNA (pJC156; chloramphenicol^R^) was added immediately prior to electroporation. Following electroporation, cells were resuspended in 1 ml THY and incubated for 1 h at 37 °C with 5% CO_2_. Cells were then serial diluted and plated onto THY or THY that contained 4 μg/ml chloramphenicol. The transformation frequency was determined by comparing the amount of transformants vs. the control cultures that were electroporated without the addition of DNA and plated onto THY that contained no antibiotic.

## Electronic supplementary material

Supplemental data

## Data Availability

The coordinates and structure factors for paratox MGAS315 (code 6CKA) have been deposited to the Protein Data Bank, Research Collaboratory for Structural bioinformatics, Rutgers University, New Brunswick, NJ (www.rcsb.org).
