# Supplementary material for: The conserved mosaic prophage protein paratox inhibits the natural competence regulator ComR in Streptococcus
Source: Sci Rep. 2018 Nov 8;8:16535. doi: 10.1038/s41598-018-34816-7 (PMC6224593; doi:10.1038/s41598-018-34816-7)
Supplement: Supplementary file 1 — Supplemental data [file 41598_2018_34816_MOESM1_ESM.pdf]

The conserved mosaic prophage protein paratox inhibits the natural competence regulator ComR in *Streptococcus*

Lauren Mashburn-Warren[1], Steven D. Goodman[1], Michael J. Federle[2], and Gerd Prehna[3]*

[1]Center for Microbial Pathogenesis, The Research Institute at Nationwide Children's Hospital, Columbus, Ohio, USA

[2]Department of Medicinal Chemistry and Pharmacognosy, Center for Biomolecular Sciences, University of Illinois at Chicago, Chicago, Illinois, USA

[3]Department of Microbiology, University of Manitoba, Winnipeg, Manitoba, Canada

*Corresponding author: gerd.prehna@umanitoba.ca

Supplemental Figures

Table S1. Strains, Plasmids, and Oligos used in this study.

Strain/Plasmid/Oligo	Description	Source
**Strains**		
MGAS315	*S. pyogenes* isolate	
MGAS8232	*S. pyogenes* isolate	
MGAS5005	*S. pyogenes* isolate	
M1 SF370	*S. pyogenes* isolate	
MW332	MGAS315 with pWAR285; Erm^R	This study
MW333	MGAS8232 with pWAR286; Erm^R	This study
MW393	MGAS5005 Δ*prx3* (M5005_spy1414); Kan^R	This study
MW407	MW393 with pWAR205	This study
MW405	M1 SF370 Δ*prx*; Cm^R	This study
MW406	MW405 with pWAR205	This study
UA159	*S. mutans* isolate	
MW30	UA159 with pWAR312; Erm^R	42
MW48	MW30 with pJC156; Erm^R	This study
MW49	MW30 with pWAR362; Erm, Cm^R	This study
MW50	MW30 with pWAR322; Erm, Cm^R	This study
MW51	MW30 with pWAR323; Erm, Cm^R	
**Plasmids**		
pFED760	Shuttle vector pGH9-ISS1 deleted for ISS1 element, temp sensitive; Erm^R	10
pWAR303	Heat resistant derivative of pGH9-ISS1 derivative carrying a 2,222-bp fragment with *luxAB* inserted between PstI and NotI sites; Erm^R	10
pWAR285	pWAR303 derivative carrying a 300-bp fragment with MGAS315 P_{prx} between the SalI and PstI sites; Erm^R	This study
pWAR286	pWAR303 derivative carrying a 300-bp fragment with MGAS8232 P_{prx} between the SalI and PstI sites; Erm^R	This study
pWAR205	pWAR303 derivative carrying a 362-bp fragment with P_{ssb} between the SalI and PstI sites; Erm^R	10
p7INT	Shuttle suicide vector that integrates at the streptococcal bacteriophage T12 *attB* site; Erm^R	43<br>42
pWAR312	Integrative derivative of p7INT carrying P_{sigX}-*luxAB* between the XbaI and BamHI sites; Erm^R	This study
pJC156	pFED760 derivative with *ermB* replaced with *cat*; Cm^R	
pWAR 362	pJC156 derivative carrying a 514-bp fragment containing the MGAS5005 M5005_spy1414 ORF and promoter region between the EcoRI and XhoI sites; Cm^R	This study<br>This study
pWAR322	pJC156 derivative carrying a 563-bp fragment containing the MGAS315 spyM3_1300 ORF and promoter region between the NotI and SalI sites; Cm^R	This study
pWAR323	pJC156 derivative carrying a 507-bp fragment containing the MGAS8232 spyM18_1444 ORF and promoter region between the NotI and SalI sites; Cm^R	
pWAR331	pET-21a derivative carrying MGAS315 *prx* (spyM3_1300) between the NdeI and XhoI sites; Amp^R	This study
pWAR349	pFED760 derivative containing *Kan* and DNA fragments flanking *prx3* to create insertion mutant; Erm^R, Kan^R	This study
pWAR368	pET-15b derivative carrying *S. mutans* UA159 *comR* between the NdeI and XhoI sites; Amp^R	This study
pWAR377	pFED760 derivative containing *cat* and DNA fragments flanking the M1 SF370 prx to create insertion mutant; Erm^R, Cm^R	This study
**Oligos**		

5005 prx3 US F	GCGTG**GCGGCCGC**TCGTCAATAAATCCATCAGATGATA	This study
5005 prx3 US R	GCGTG**CTGCAG**TGTTAGCATAGATTACCTCCTTATC	This study
5005 prx3 DS F	GCGTG**GTCGAC**GAATTGGAGTAACATTACCCGTCAA	This study
5005 prx3 DS R	GCGTG**CTCGAG**TTATAGGCAGACTTTAATGATTCTGTCAC	This study
M1 prx US F	GCGTG**GCGGCCGC**ATCTTCTTCGTTTTGAGTTGAATCTAGCA	This study
M1 prx US R	GCGTG**GTCGAC**GTATGTTAGCATTTTTACCCTCCTACTTA	This study
M1 prx DS F	GCGTG**CTGCAG**CTTTCGAGATAAGTATTTTCTTTGCCC	This study
M1 prx DS R	GCGTG**CTCGAG**GCCCGTCAATTGGAACAGCTT	This study
315 Pprx F	GCGTG**GTCGAC**CTCTTGCGAGATTGTTAGTCCAAGAAATGTCA CTAAAAC	This study
315 Pprx R	GCGTG**CTGCAG**ATTTCATCCTCCTACCTATCTATTC	This study
8232 Pprx F	GCGTG**GTCGAC**TGAGAATAGCTATTAAAATAACAAC	This study
8232 Pprx R	GCGTG**CTGCAG**TTTTACCCTCCTACTTATTTATTCG	This study
5005 prx F	GCGTG**GAATTC**ATAACGTAGCTAGAATGGTC	This study
5005 prx R	GCGTG**CTCGAG**AAAAAATAAAAATAAACGGGTGATTGACG G	This study
315 prx F	GCGTG**GCGGCCGC**TACCTCTTGCGAGATTGTTAGTCCAA	This study
315 prx R	GCGTG**GTCGAC**GTATAGATAAAACTCCTAAAATTGTGGC	This study
8232 prx F	GCGTG**GCGGCCGC**TGATTTAGACATATCCATGTCCTCC	This study
8232 prx R	GCGTG**GTCGAC**CTATGTAAGTTTTAAACTTATGCCCCC	This study
315 prx pET F	GCGTG**CATATG**TTATATATAGATGAGTTTAAAG	This study
315 prx pET R	GCGTG**CTCGAG**TTTGTCTAATTCCACCATCACTTCT	This study
UA159 comR pET F	GCGTG**CATATG**TTAAAAGATTTTGGGAA	This study
UA159 comR pET R	GCGTG**GGATCC**TTATGTCCCGTTCTGAGAAT	This study
UA159 PsigX F	TTGAATCGGGTAGCATATAA	This study
FAM-UA159 PsigX R	5’FAM-CTATTACGATGACCTCCTTT	This study
UA159 PcomS F	AAGCAGGTAGACTGCCTTCCATTGG	This study
FAM-UA159 PcomS R	5’FAM-CCTGTTATTCTCCTTTCTTT	This study
Kan F	GCGTG**CTGCAG**GGCAAGGCATAGGCAGCGCGCTTATCAAT	This study

Kan R	GCGTG**GTCGAC**CAGAAAAGATTAGATGTCTAAAAAGCTTG	This study
Cat F	GCGTG**GTCGAC**GATGAAAATTTGTTTGATTT	This study
Cat R	GCGTG**CTGCAG**TTATAAAAGCCAGTCATTAG	This study

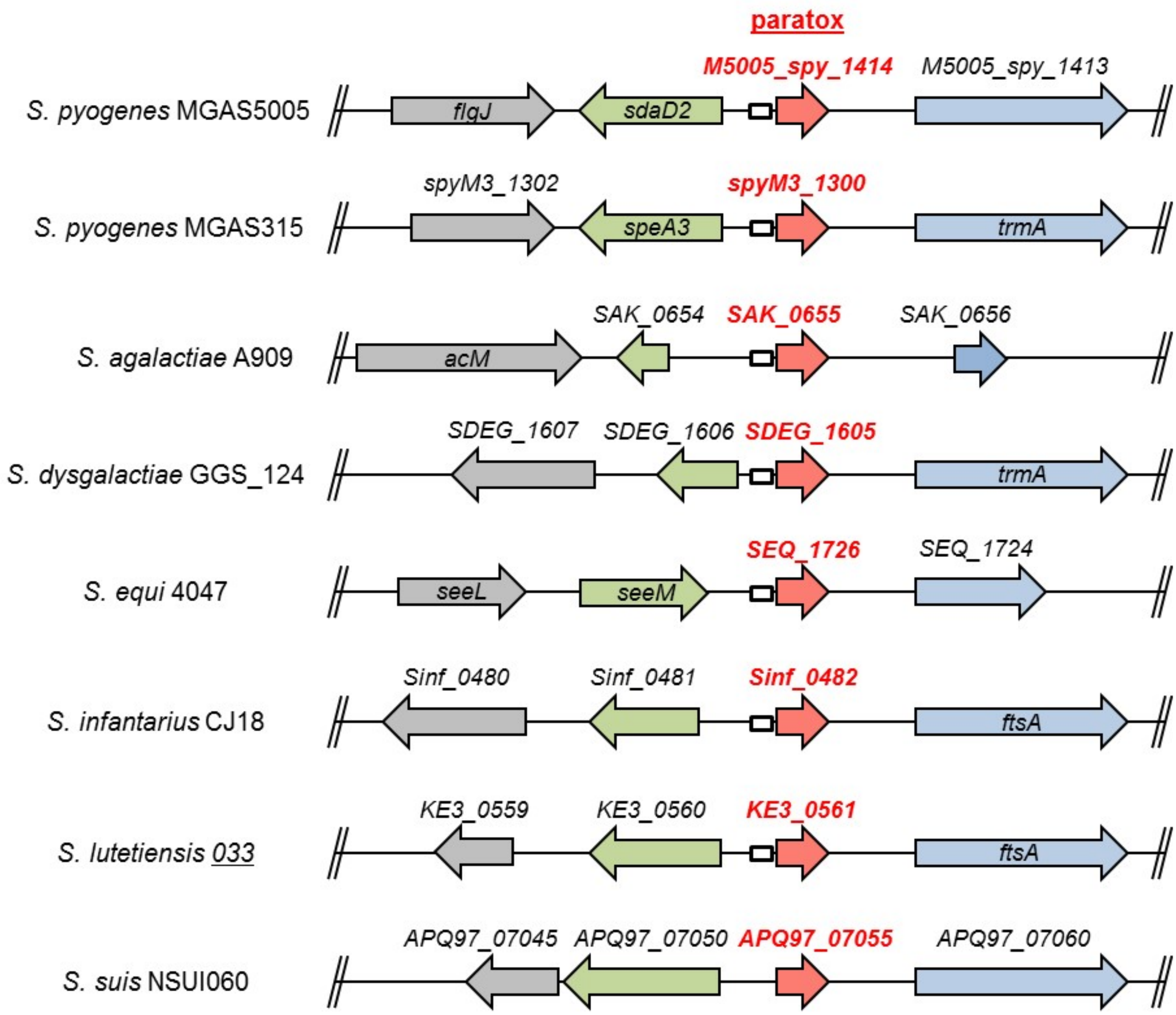

Figure S1: Paratox species locus. Alignment of prophage terminal ends in diverse *Streptococcus* species, indicating conservation of paratox orthologs (red) and adjacent toxin genes (green). Black vertical bar indicates the *attR* prophage terminus. Conserved CIN-boxes (TACGAATA, marked as open black rectangles) were located proximally to *prx* in every instance with the exception of *S. suis*.

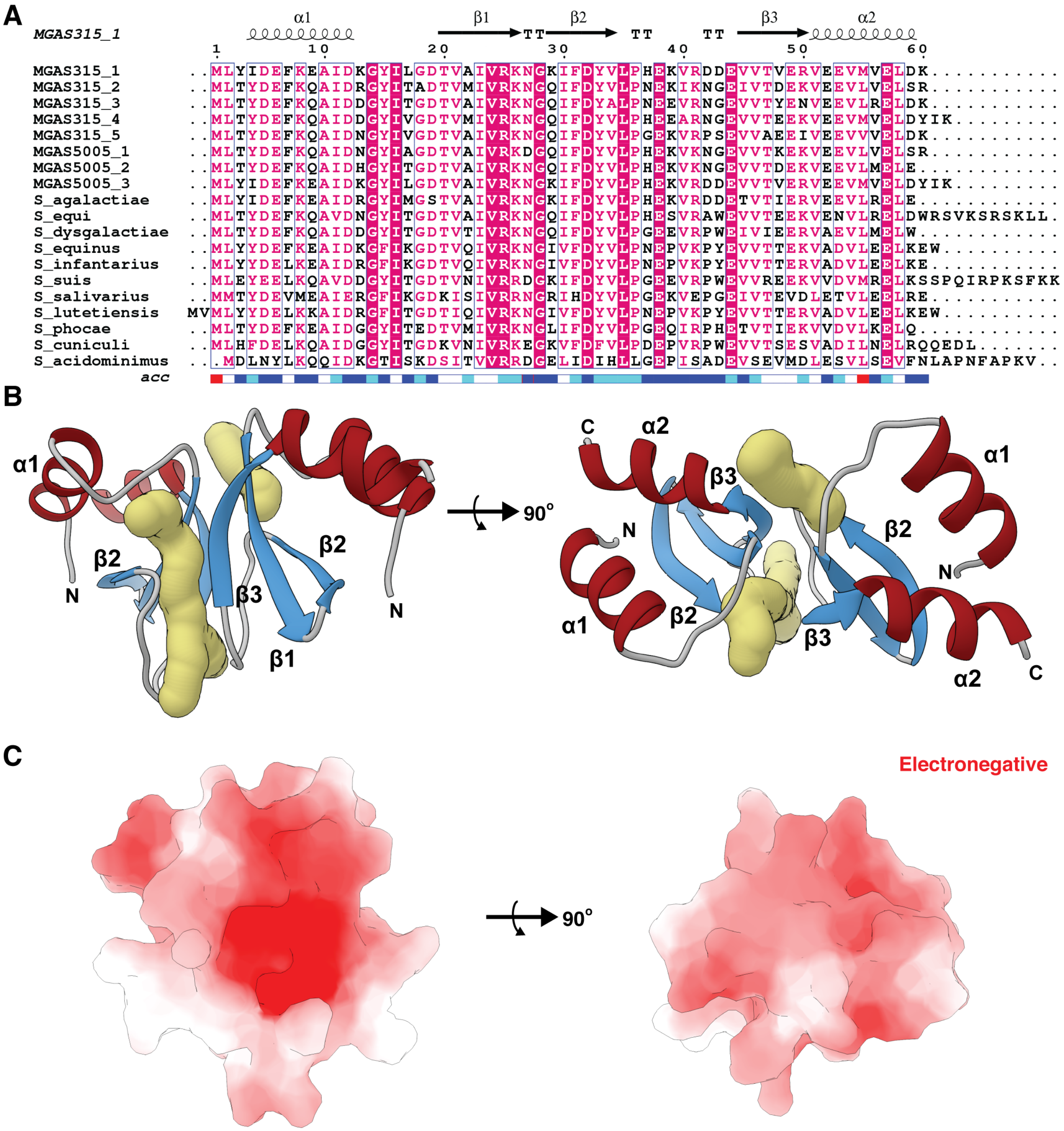

Figure S2. Paratox multisequence alignment and crystallographic dimer interface analysis. (A) Paratox sequences labeled by species paired down to 17 from 114 Consurf sequences. Magenta box indicates complete conservation and magenta letter homologous residues. The top indicates secondary structure elements and the bottom solvent accessible residues (dark blue), partially exposed (light blue) and buried (white). (B) Large solvent channels between the crystallographic dimer as calculated by Moleonline 2.0 are shown in yellow. (C) Molecular surface of Prx colored by electrostatic potential using the Adaptive-Poisson-Boltzman solver at pH 7.5 (PARSE) and contoured from -10 (red) to 10 kT/e (blue). Clustal omega and Espript3 were used to create the sequence alignment. Molecular graphics were drawn using UCSF Chimera (https://www.rbvi.ucsf.edu).

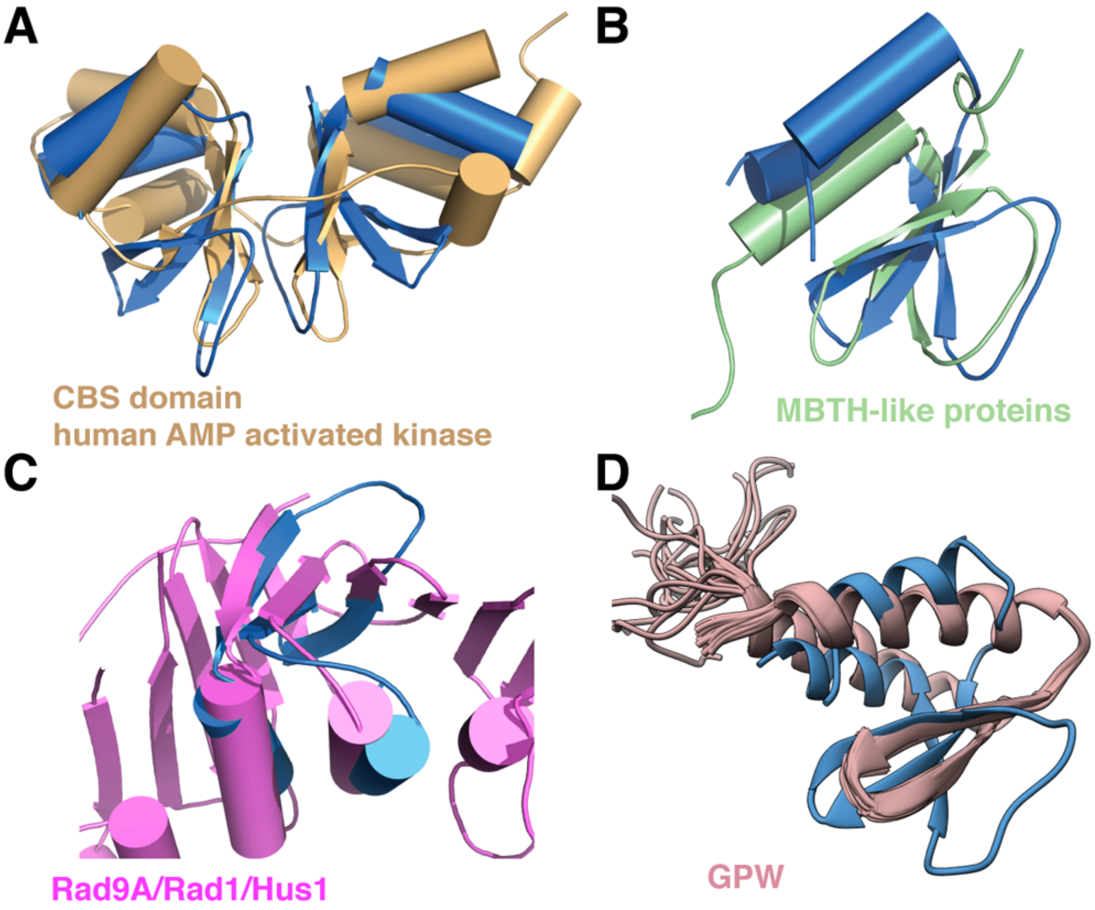

Figure S3. Structural relatives of paratox. Shown here are four examples of distant structural relatives isolated from homology searches using the Dali server and PDBefold. In each panel paratox is blue. (A) Alignment of the crystallographic dimer to a CBS domain (PDB code: 2UV5). (B) paratox shows minor homology to MBTH-like proteins such as YBDZ (PBD code: 5ja1). (C) Alignment with one of the repeating units of Rad9A/Rad1/Hus1 complex (human) involved in DNA repair (3g65). (D) paratox shows some homology to GPW of Bacteriophage Lambda (PDB code: 2L6Q). Molecular graphics were drawn using UCSF Chimera (https://www.rbvi.ucsf.edu) and Pymol (https://pymol.org).
